# Identifying molecular signatures of post-traumatic stress disorder vulnerability and progression in a longitudinal study: a study protocol

**DOI:** 10.3389/fpsyt.2025.1584583

**Published:** 2025-07-30

**Authors:** Federico Suprani, Pasquale Paribello, Giulia Federica Mancini, Maria Morena, Marco Pinna, Federica Pinna, Martina Contu, Caterina Visioli, Fabio Medas, Gian Luigi Canu, Federico Cappellacci, Pietro Giorgio Calò, Gabriele Finco, Salvatore Sardo, Monica Maria Francesca Puligheddu, Ernesto D’Aloja, Claudia Pisanu, Alessio Squassina, Donatella Congiu, Gian Marco Leggio, Mirko Manchia, Patrizia Campolongo

**Affiliations:** ^1^ Section of Psychiatry, Department of Medical Sciences and Public Health, University of Cagliari, Cagliari, Italy; ^2^ Unit of Psychiatry, University Hospital Agency of Cagliari, Cagliari, Italy; ^3^ Department of Physiology and Pharmacology, Sapienza University of Rome, Rome, Italy; ^4^ Department of Biomedical and Biotechnological Sciences, University of Catania, Catania, Italy; ^5^ Neuropharmacology Unit, Istituto di ricovero e cura a carattere scientifico (IRCCS) Fondazione Santa Lucia, Catania, Italy; ^6^ Centro Bini for Mood Disorders, Cagliari, Italy; ^7^ Department of Surgical Sciences, University of Cagliari, Cagliari, Italy; ^8^ Department of Medical Sciences and Public Health, University of Cagliari, Cagliari, Italy; ^9^ Unit of Neurology, Department of Medical Sciences and Public Health, University of Cagliari, Monserrato, Cagliari, Italy; ^10^ Unit of Neurology, University Hospital Agency of Cagliari, Monserrato, Monserrato, Cagliari, Italy; ^11^ Section of Legal Medicine, Department of Medical Sciences and Public Health, University of Cagliari, Cagliari, Italy; ^12^ Section of Neuroscience and Clinical Pharmacology, Department of Biomedical Sciences, University of Cagliari, Cagliari, Italy; ^13^ Section of Pharmacology, Department of Biomedical and Biotechnological Sciences, University of Catania, Catania, Italy; ^14^ Department of Pharmacology, Dalhousie University, Halifax, NS, Canada

**Keywords:** PTSD, stress, trauma, omics, cannabinoids, microRNA

## Abstract

Post-Traumatic Stress Disorder (PTSD) is a highly debilitating psychiatric disorder, which develops in a subset of trauma-exposed individuals. Patients with PTSD fail to extinguish fear responses to no-longer dangerous stimuli and develop enduring experiences of fear and anxiety. To advance the understanding of PTSD neurobiology, longitudinal and comprehensive clinical and molecular data are needed. Here we present the protocol of the project “Reli€ving-PTSD” aiming at identifying the molecular signatures of PTSD vulnerability and disease progression in a longitudinal study in humans. The molecular signature will be based on the analysis of the endocannabinoid (eCB) system, as well as miRNome and transcriptome profiles. The study will recruit 60 participants hospitalized in the Intensive Care Unity of the University Hospital Agency of Cagliari. Participants will be eligible for this study if they are: 1) between 18 and 65 years old; 2) able to provide written informed consent. We plan to recruit 30 patients with a diagnosis of PTSD or Acute Stress Disorder (ASD) according to DSM-5 and 30 patients without either diagnosis. Exclusion criteria are: 1) history of traumatic brain injury; 2) current and/or lifetime diagnosis of psychiatric disorders other than PTSD/ASD; 3) current and/or lifetime diagnosis of substance use disorder; 4) presence of severe neurological or medical morbidity. These stringent eligibility criteria will reduce the confounding effect of comorbidities, as molecular alterations of the eCB system have been associated to several psychiatric disorders. This research addresses critical gaps in PTSD management. The outcomes are anticipated to significantly advance scientific knowledge, inform clinical practices, and benefit public health by reducing the societal and economic burden of PTSD through improved precision medicine-based prevention and treatment strategies. The study was reviewed and approved by the Ethics Committee of the Region of Sardinia (Prot. CE/2023_014) and funded by the European Union - Next Generation EU - NRRP M6C2 - Investment 2.1 Enhancement and strengthening of biomedical research in the NHS.

## Introduction

Post-Traumatic Stress Disorder (PTSD) is a highly debilitating psychiatric disorder, which develops in a subset of trauma-exposed individuals. Patients with PTSD fail to extinguish fear responses to no-longer dangerous stimuli and develop enduring experiences of fear and anxiety (1). Estimates suggest that the cross-national lifetime prevalence of PTSD in the population is up to 3.9%, with roughly half of the cases being persistent (2). According to naturalistic long-term studies, only the minority of patients achieves a stable recovery within 3–7 years (3). The economic burden of PTSD is due to both the direct health-care costs and the high rates of associated unemployment and disability (4).

Types of traumatic events associated with PTSD onset include sexual trauma, physical assault, witnessed violence, natural disaster, terrorism, combat, violent death of a loved one, motor vehicle collision, and life-threatening illness (5). In fact, in a UK prospective multicenter study, the prevalence of PTSD among Intensive Care Units (ICU) survivors was 22% (6). Given the heterogeneity of the traumatic events associated with PTSD onset, the exposure to potential triggers appears to be relatively frequent in the population. According to the Italian National Institute of Statistics (ISTAT), in the first semester of 2023 more than 100,000 people were injured in traffic collisions across the country (7). Moreover, large-scale traumatic events have happened in the past few years, exposing a high number of individuals to the potential psychological consequences. For instance, high levels of PTSD symptoms were measured in the Italian general population during the COVID-19 pandemic, especially among women and younger generations (8). More recently, data on the Ukrainian population have shown a high prevalence of PTSD symptoms (> 50%) in the 9–12 months after the start of the February 2022 Russian invasion (9).

While the symptom burden can be substantial-particularly in high-risk populations exposed to cumulative or prolonged trauma-the lifetime prevalence of full-syndrome PTSD in the general population remains lower; for instance, it is estimated at 6.1% in the United States (10). Epidemiological evidence has consistently shown that only a minority of trauma exposed individuals go on to develop PTSD (11–13). The neurobiological mechanisms underpinning the individual variation in vulnerability to PTSD development following trauma are still unclear (14). Different biological systems have been investigated, such as those involved in neuroplasticity and immunoregulation (15). More recently, the endocannabinoid (eCB) system has received attention in the PTSD research field, given its modulatory effect on memory consolidation, retrieval and extinction (16). There is a large amount of data in animal models supporting the eCB system role in regulating fear and stress responses (17, 18). The fatty acid-derived signaling molecule Anandamide (AEA), the first discovered endocannabinoid, was shown to facilitate fear extinction in mice by activating the Cannabinoid receptor 1 [CB1 (19, 20)]. When research on the eCB system in fear responses was extended to humans, consistent results were found, mostly on healthy subjects. Three different human studies replicated the association between enhanced fear extinction and a hypofunctional genetic variation in the fatty acid amide hydrolase (FAAH), an enzyme that catabolizes AEA (21–23). Positive results in genetics research were also published by Heitland et al. (24), who found that A/A carriers of rs2180619 (a polymorphism located in the promoter region of CB1), exhibited impaired fear extinction. In line with preclinical and healthy volunteers’ literature, individuals with PTSD were shown to have a reduction in circulating endocannabinoids levels (25, 26).

A recent study identified the amygdala-projecting medial prefrontal cortex (mPFC) neurons as a potential neural basis for the effect of the eCB system on fear extinction (27). The amygdala is a primary hub in the brain that regulates the processing of aversive memories, by integrating information from different cortical brain regions (28). Among these regions, the mPFC was shown to exert top-down control of negative emotions over the amygdala in mice (29). Findings in patients with PTSD suggest that the interaction between the mPFC and the amygdala becomes dysfunctional, resulting in the heightened responsivity of the latter (30). Several recent studies with different designs support this hypothesis. For instance, neurosurgical patients with ventral mPFC lesions exhibited potentiated amygdala responses to aversive images compared to neurologically healthy controls (31). Consistently, Feng et al. (32), showed that the resting-state functional connectivity between the ventral mPFC and the amygdala is positively correlated with fear extinction after fear reminder in healthy subjects. Overall, numerous lines of evidence point toward a potential role of the eCB system in PTSD vulnerability following trauma, possibly via altered top-down control on the amygdala.

To advance the understanding of PTSD neurobiology, longitudinal and comprehensive clinical and molecular data are needed. The project “*Reli€ving-PTSD”* aims to identify the molecular signatures of PTSD vulnerability and disease progression in a longitudinal study in humans. The development of high-throughput methods has recently facilitated the detection of differentially regulated biological molecules via omics analysis, enabling the generation of new hypotheses on dysregulated pathways (33). MiRNomics and transcriptomic research has been applied to central and peripheral tissues from both humans with PTSD and animal models (34–36). Findings include over-representation of several miRNA- families (34, 36) and over-expression of genes involved in inflammatory response and immune signaling (37–39). In *Reli€ving-PTSD*, we will recruit patients hospitalized in ICU and followed-up for one year. We will: (1) collect longitudinal data on biochemical markers of the eCB and stress systems; (2) perform a transcriptome and miRNome analysis. Through this longitudinal approach, we aim to further explore PTSD neurobiology, possibly unraveling new targets for precision medicine prophylactic and/or therapeutic interventions.

## Methods and analysis

### Study design

*Reli€ving-PTSD* is a longitudinal project designed to identify molecular markers of PTSD vulnerability and progression. ICU survivors will be assessed between 3 and 60 days after ICU discharge (T0) and after 1 year (T1). Assessment will consist of PTSD symptoms evaluation with standardized questionnaires and blood samples collection. Blood/plasma will be collected for identification of biochemical markers of the eCB and stress systems, as well as for miRNome and transcriptome analyses, to uncover early and late post-trauma alterations associated with vulnerability or resilience to the development of PTSD. Transcriptome/miRNome data will be validated by real-time PCR.

### Aims and hypotheses

#### Aim 1

Characterization of the neuroendocrine determinants of PTSD vulnerability and progression in human patients.

#### Hypothesis 1

Patients exposed to trauma who develop PTSD symptoms have early (T0) and late (T1) distinct eCB and stress systems molecular signatures compared to asymptomatic trauma-exposed patients sampled at the same time points.

#### Aim 2

Identification of miRNomics and transcriptomics signatures associated with PTSD vulnerability and progression in human patients.

#### Hypothesis 2

Patients exposed to trauma who develop PTSD symptoms have early (T0) and late (T1) distinct miRNomics and transcriptomics signatures compared to asymptomatic trauma-exposed patients sampled at the same time points.

### Clinical population

The study will recruit 60 participants hospitalized in the ICU of the University Hospital Agency of Cagliari. The sample size is adequately powered (Beta>90%) given previous data showing an effect size (F) of 3.2 in differences between baseline and six-eight months assessment of eCB metabolites (40). Participants will be eligible for this study if they are: 1) between 18 and 65 years old; 2) able to provide written informed consent. We plan to recruit 30 patients with a diagnosis of PTSD or Acute Stress Disorder (ASD) according to DSM-5 and 30 patients without either diagnosis. We aim to enroll a gender-balanced sample, with approximately equal numbers of male and female participants in each group. Exclusion criteria are: 1) history of traumatic brain injury; 2) current and/or lifetime diagnosis of psychiatric disorders other than PTSD/ASD; 3) current and/or lifetime diagnosis of substance use disorder; 4) presence of severe neurological or medical morbidity. These stringent eligibility criteria will reduce the confounding effect of comorbidities, as molecular alterations of the eCB system have been associated to several psychiatric disorders (41). All participants will provide written informed consent.

### Procedures and measures

The following paragraph outlines all the procedures and the outcome measures, organized by study section and type of assessment. A summary is provided in **Table 1**.

**Table 1:** List of assessment for outcome measures.

Behavioral and clinical outcome measures	Molecular outcome measures
• PCL-5 total score • CAPS-5 total symptom severity score • CAPS-5 PTSD diagnosis • CAPS-5 reexperiencing score • CAPS-5 avoidance score • CAPS-5 negative alterations in cognition and mood score • CAPS-5 hyperarousal score • CAPS-5 dissociation score	• Levels of AEA, 2-AG, OEA, PEA • Levels of Cortisol, • Total RNA-Seq • miRNA-Seq

PTSD, Post-Traumatic Stress Disorder; CAPS-5, Clinician-Administered PTSD Scale; PCL-5, PTSD Checklist for the DSM-5; 2-AG, 2-arachidonoylglycerol; AEA, N-arachidonoylethanolamine; OEA, N-oleoylethanolamine; PEA, N-palmitoylethanolamine.

Participants will be recruited 3 to 60 days after ICU discharge, based on clinical stability and the ability to provide informed consent. A patient will be considered clinically stable if their condition is compatible with outpatient care. Clinical evaluation, plasma and blood sampling will be done concurrently at baseline (T0) and at a 12-months follow-up (T1).

#### Clinical assessment

To evaluate the presence and severity of PTSD symptoms we will employ: 1) the Italian version of the PTSD Checklist for the DSM-5 (PCL-5, 42); and 2) the Clinician-Administered PTSD Scale for DSM-5 (CAPS-5, 43). The PCL-5 is a self-report questionnaire composed of 20 items that are scored on a 5-point Likert scale. It was primarily developed for PTSD screening and longitudinal symptoms monitoring (42). The Italian version of the PCL-5 has good validity and reliability (44). The CAPS-5 is a structured clinical interview that represents the gold standard in PTSD assessment and diagnosis according to DSM-5 (43). For the study the past-month version of the CAPS-5 will be administered by a trained psychiatrist. The first assessment (T0) will be made 3-to-60 days after ICU discharge. The scales administered will allow the dimensional assessment of post-traumatic stress symptoms and the possible categorical diagnosis of PTSD or ASD according to DSM-5 criteria. Several studies have assessed PTSD symptoms in ICU survivors in the first weeks after hospital discharge and found that 5-45% of patients were already showing symptoms (45–48). The same clinical assessment will be repeated at a 12-months follow-up on the whole sample (T1), to identify longitudinal trajectories of PTSD symptoms and distinguish resilient participants (RES) from susceptible ones (SUS, defined as participants with a PTSD diagnosis according to DSM-5 criteria one year after trauma).

#### Molecular analysis

We will assess levels of biochemical markers of the eCB and stress systems (e.g. circulating endocannabinoid levels and cortisol) and then we will perform transcriptome and miRNome analysis. Blood/plasma samples will be immediately stored at -80°C after collection. PAXgene Blood RNA Tube (QIAGEN N.V., Hilden, Germany) will be used to collect blood for RNA analysis. After the identification of alterations, we will proceed to validate them by using real-time PCR. Single-stranded cDNA will be synthesized from total RNA using the SuperScript III Reverse Transcriptase kit (Thermo Fisher Scientific, Waltham, MA, USA). RT-qPCR reactions will be conducted on a 7900HT Fast Real-Time PCR System (Applied Biosystems, Life Technologies, Carlsbad, CA, USA). RNA isolation, cDNA synthesis and PCR will be performed in the plasma of human participants.

### Data analysis

Statistical analyses will be performed using IBM SPSS Statistics for Windows, Version 27 (IBM Corp., Armonk, NY, USA), R software (Version 1.2.5042). Between-groups comparisons of neuroendocrine molecular markers, transcriptome and miRNome profiles will be tested with ANOVA and MANOVA as appropriate *Post-hoc* Tukey’s test will be applied as needed. Normality of data distribution will be ascertained through Shapiro-Wilk test. In the unlikely case of not normally distributed data, Kruskal-Wallis test will be applied. To test the association between PTSD symptoms and biological dependent variables, we will use multivariate correlation plots generated using the corrplot package in R, applying Spearman correlation coefficients. Regression analyses will be used to model the interactions among gender, trauma type, and circulating eCB levels in relation to PTSD symptom severity. P- values of less than 0.05 will be considered statistically significant.

### Data management and confidentiality

Personal data protection for human participants will be ensured in accordance with the European Union General Data Protection Regulation (GDPR). Each participant will be de-identified through pseudonymization within both the paper-based Data Collection Form and the electronic database using a unique progressive number. Only the Principal Investigator and the authorized medical personnel (who are bound to professional confidentiality according to the Italian Law) will have access to the code allowing for re-identification. Participants will have the right to withdraw their consent to collect, use, or publish their data at any time without the need to provide further explanations. In compliance with the Article 13 of the GDPR (2016/679), the results of this study may be published on scientific journals while ensuring the protection of the participants’ personal data.

## Discussion

PTSD is a chronic and debilitating condition characterized by significant morbidity and an overwhelming burden on healthcare systems, largely due to the absence of effective treatments (3, 4). The *Reli€ving-PTSD* project aims to identify molecular biomarkers that influence vulnerability, resilience, and the progression of PTSD across sexes, by integrating neuroendocrine profiling with transcriptomic and miRNA analyses. Our multidisciplinary approach will be valuable in enhancing the understanding of the disorder and fostering innovative strategies in prevention and treatment. A key focus of the project is the identification of biological markers associated with PTSD vulnerability, enabling the early identification of individuals who may benefit from timely interventions following trauma exposure. Additionally, the discovery of protective factors will provide critical insights into the mechanisms underlying resilience, creating opportunities for the development of prophylactic strategies.

The project has important strengths: (i) the use of an unbiased ‘omics’ approach allowing the discovery of novel, unexplored targets, paving the way for the development of future treatments; (ii) its longitudinal design, enabling the identification of early predictors of PTSD following ICU treatment. Beyond its focus on PTSD, the framework of *Reli€ving-PTSD* holds promise for application to other trauma- and stress-related mental health conditions, expanding its potential clinical and societal impact.

Despite its strengths, the present study has several limitations. First, the sample size limits the possibility of conducting fully powered subgroup analysis regarding hormonal status in female participants (e.g., before/after menopause), which would be of particular interest as evidence point towards a strong influence of ovarian hormones in the eCB system (49). Second, the heterogeneity of reasons leading to ICU admission may contribute to variability in PTSD risk. To address this, trauma type will be accounted for in the statistical analysis. Third, the risk of attrition at the 12-months follow-up might reduce the statistical power of our longitudinal analysis. We plan to mitigate it by close collaboration with general practitioners. Fourth, endocannabinoid concentrations in biological fluids often exhibit significant variabilities across experimental conditions, thus posing a challenge in the reliability of molecular analysis (50). To reduce the risk of bias, we will: (a) use plasma as the biological matrix, due to its higher endocannabinoid concentrations and improved signal-to-noise ratio; (b) collect all blood samples between 9:00 and 11:00 AM to minimize variability due to circadian fluctuations (51); (c) process samples rapidly by placing blood on ice, centrifuging within 15 minutes and storing plasma at -80°C to minimize ex-vivo synthesis by blood leukocytes and platelets and to maximize the stability of endocannabinoids levels over time (52). Fifth, the lack of pre-trauma and genotypic data prevents a clear distinction between inherited and acquired molecular alterations. Our design focuses on identifying post-trauma biological changes and their longitudinal association with psychopathological outcomes and do not directly address whether the molecular signatures observed are predisposing traits or consequences of trauma exposure. Finally, the highly specific nature of our ICU sample will limit the generalizability of our findings to other trauma-exposed populations. Future replication studies will be required to enhance external validity of our results.

In conclusion, this research addresses critical gaps in PTSD management. The outcomes are anticipated to significantly advance scientific knowledge, inform clinical practices, and benefit public health by reducing the societal and economic burden of PTSD through improved precision medicine-based prevention and treatment strategies.
